# Editorial: Structural variation of the chloroplast genome and related bioinformatics tools

**DOI:** 10.3389/fpls.2024.1341528

**Published:** 2024-04-08

**Authors:** Linchun Shi, Gang Zhang, Tapan Kumar Mohanta, Weijun Kong, Baozhong Duan

**Affiliations:** ^1^ State Key Laboratory for Quality Ensurance and Sustainable Use of Dao-di Herbs, Institute of Medicinal Plant Development, Chinese Academy of Medical Sciences and Peking Union Medical College, Beijing, China; ^2^ College of Pharmacy, Shaanxi University of Chinese Medicine, Xianyang, Shaanxi, China; ^3^ Natural and Medical Sciences Research Center, University of Nizwa, Nizwa, Oman; ^4^ School of Traditional Chinese Medicine, Capital Medical University, Beijing, China; ^5^ College of Pharmaceutical Science, Dali University, Dali, China

**Keywords:** chloroplast genome, pseudogenes, gene losss, population study, bioinformatics tools

## 1 Introduction

Chloroplast genomes (plastomes) serve as crucial data sources for plant phylogenetic reconstruction and molecular identification. However, comprehensive studies on the assembly, annotation, and in-depth analysis of plastomes exhibiting distinct features such as pseudogenes, gene losses, duplications, rearrangements, widespread intra-individual polymorphisms, and large-scale horizontal gene transfer are still lacking. For instance, the plastomes of various saprophytic and parasitic plants have been observed to undergo a significant loss of photosynthesis-related genes. High-quality assembly, annotation, and in-depth analysis of these plastomes remain major challenges. In addition, more powerful bioinformatics tools are needed to facilitate large-scale plastome studies (Shi et al., 2019). This Research Topic on “Structural Variation of the Chloroplast Genome and Related Bioinformatics Tools” contains ten research articles, emphasizing reliable plastome studies with complex structures or plastomes for population studies, in addition to bioinformatics tools for in-depth analysis of these plastome data. These articles provide a crucial scientific basis for analyzing the structural characteristics of plastomes, studying phylogenetic and genetic evolution, developing new molecular markers, and exploiting bioinformatics tools.

## 2 Results

### 2.1 Characterization and in-depth analysis of plastomes with special structures

The plastome of non-photosynthetic plants often undergoes gene loss (Mohanta et al., 2020), pseudogenic transformation, and rearrangement. Orchidacea is a typical taxonomic group, and their plastomes underwent gene loss and gene pseudogenization (Kim et al., 2020). Zhou et al. decoded the plastome of *Galeola lindleyana* in the Vanilloideae subfamily, a heterotrophic orchid plant, and compared it with previously published photosynthetic and heterotrophic orchid plastomes. The length of the *G. lindleyana* plastome has been reduced to 100,749 bp, while still retaining its typical quadripartite structure. In half-heterotrophs, the reduced photosynthetic function begins with the loss of non-essential or stress-related genes, such as *ndh* genes, followed by pseudogenization and the loss of major photosynthesis-related genes (such as *pet*, *psa* and *psb* genes) and plastid-encoded polymerases.

### 2.2 Large-scale comparative plastome studies for populations

In this Research Topic, there are eight research articles that present comparative analysis of plastomes of plants such as *Solanum lycopersicum* L., *Pseudostellaria heterophylla* (Miq.) Pax, *Corydalis platycarpa* (Maxim. ex Palib.) Makino, *Aristolochia* L., *Phyllanthus* L., *Kandelia* Wight & Arn., *Artemisia* L., and *Paraboea* (Clarke) Ridley. These articles provide valuable information for molecular identification and phylogenetic analysis.

Plastomes can provide distinguishing features and more molecular markers to help identify closely related species and even as super barcodes for accurate identification of plants and germplasm resources (Gao et al., 2023). Lan et al. analyzed 15 plastomes from 5 *Artemisia* species, including 12 newly sequenced genomes. Four hotspot regions and 189-192 SSR molecular markers were identified, which can serve as potential DNA barcodes for further studies on *Artemisia* species. Fang et al. *de novo* assembled and characterized the complete plastomes of nine species of the genus *Phyllanthus*. The authors highlighted three highly variable regions (*trnS-GCU-trnG-UCC*, *trnT-UGU-trnL-UAA*, and *petA-psbJ*) that may be useful as potential molecular markers for identifying *P. urinaria* and its adulterants. Wang et al. sequenced and analyzed the plastomes of 29 tomato germplasm lines. Among the screened SNP markers, those localized to segments of the *ndhH* gene and the *ndhK-ndhC-trnV-UAC* gene spacer region could be used for interspecific identification. The developed SNP markers can be used to analyze genetic diversity and population structure at the plastome level and to develop functional markers associated with traits such as male sterility. Bai et al. sampled 11 species of *Aristolochia* collected from distinct habitats in China and sequenced their complete plastomes. Their analysis of simple sequence repeats (SSRs) was able to identify potential molecular polymorphic markers for analyzing the genetic diversity and structure of *Aristolochia* populations in the future. Highly variable regions would provide candidate markers for *Aristolochia* species identification studies. Zhang et al. collected 17 P*. heterophylla* plant samples with remarkable phenotypic characteristics and obtained their plastome sequences. The authors verified that plastomes could elucidate the relationship among closely related cultivated materials and provide useful information for the development of new, highly polymorphic, and informative molecular makers.

In addition, other articles provide important insights into adaptive evolution and phylogeny, and offer significant guidance for future research on plant evolution and conservation. Raman et al. sequenced the plastome of *C. platycarpa* and conducted wide-scale comparative studies using publicly available data from 20 *Corydalis* plastomes. The results revealed extensive genome rearrangement and IR expansion, events that evolved independently in the *Corydalis* species. The divergence time of the *ndh* gene in the *Corydalis* sub-clade species (44.31-15.71 mya) is consistent with the uplift of the Qinghai-Tibet Plateau in the Oligocene and Miocene, and may have triggered the radiation of the *Corydalis* species during this period. In 2003, *Kandelia obovata* was identified as a new mangrove species distinct from *Kandelia candel*. Xu et al. sequenced the 25 whole plastomes of *K. obovata* (18 samples) and *K. candel* (Seven samples) for comparison. A comparative molecular simulation study of the homologous NAD(P)H dehydrogenase chain 4 (NDH-D) and ATP synthase subunit alpha (ATP-A) proteins of *K. candel* and *K. obovata* predicted that the functions of photosynthetic electron transport and ATP generation were significantly different. The results suggest that energy demand is a pivotal factor in their adaptation to different environments geographically separated by the South China Sea. Wang et al. sequenced and compared the complete plastomes of 12 *Paraboea* species from China and Vietnam. The study presents several important findings:

1. It demonstrates the strong conservation of the plastomes among *Paraboea* species.
2. It confirms the monophyletic nature of the genus. The study also reveals the impact of purifying selection on the protein-coding genes in the plastomes.
3. It emphasizes the significance of understanding the genetic mechanisms underlying plant adaptation to specific environmental conditions, particularly karst environments.

### 2.3 Bioinformatics tools for plastome annotation and analysis

Genome comparison of multiple plastomes involves many software, including some popular tools, such as mVISTA, Mauve, IRscope, etc. However, these tools are separate from each other and lack a unifying interface, making comparison analysis a time-consuming and labor-intensive task. An automated workflow for fast and accurate assembly and annotation of plastome sequences from raw whole (nuclear) genome sequencing data is needed. Chen et al. developed Plastaumatic-an automated pipeline for both the assembly and annotation of plastomes, with the ability to load whole genome sequence data with minimal manual input and, therefore a faster run time. The pipeline was demonstrated on two sets of plant sequence data: three soybean accessions and 12 potato accessions. It showed substantially faster completion than manual assembly. This automated pipeline, which includes plastid assembly and annotation is an efficient tool. In their article, Zhou et al., used the GetOrganelle (V1.7.7.0) assembly toolkit which provides fast and accurate *de novo* assembly of the organelle genome. They also used the CPGAVAS2 web server, which automatically annotates the plastome.

## 3 Perspectives

This Research Topic has conducted a series of comparative studies on plastomes, with their characterization and in-depth analysis, and on bioinformatics analysis tools. These contributions are of great value for the analysis of genomic features, phylogenetic and genetic evolution, and the development of new molecular markers. However, more research is needed on the plastomes of parasitic plants, saprophytic plants, or plants living under extreme environmental conditions, such as drought, cold, high altitude, and high soil salinity environments. For instance, during the rapid radiation and evolution of *Rhodiola* plants growing at high altitudes, the evolution of plant plastomes and their role in adaptation to extreme habitats remained largely unknown. Therefore, the study of plant plastomes under harsh environmental conditions emerges as an important research direction for the future, which will help deepen our understanding of the mechanisms behind plant adaptation to extreme environments and will provide crucial insights for future studies on plant evolution and conservation.

In addition, the plant chloroplast genome has reached a critical point. With recent advances in sequencing technology, the speed of generating chloroplast genomes has increased dramatically. The number of chloroplast genome sequences of Viridiplantae plants has increased from about 3,000 in 2019 to more than 25,000 currently. However, there are several fundamental issues that need to be addressed urgently: i, A standardized nomenclature for tRNA genes and protein-coding genes has not yet been uniformly accepted for all chloroplast genomes due to the lack of a plastid gene nomenclature committee like that for humans (https://www.genenames.org/), and this has prevented the large-scale comparative analysis of chloroplast genome data in an automated way. The proposed solution to this issue may start with the naming uniformity among several popular annotation tools, such as Geseq, CPGAVAS2, AGORA, etc., or by public databases, such as NCBI. ii, The number of chloroplast coding genes may be obscured in some taxonomic groups due to over-reliance on chloroplast genome annotation tools. The two popular annotation tools, GeSeq and CPGAVAS2, rely on a curated reference sequence database, and *de novo* annotation tools are still lacking. iii, The methods of comparative analysis of chloroplast genomes tend to be similar, and new essential tools need to be developed to facilitate the deep mining of chloroplast genome data, such as species differentiation, species expansion, and species adaptation to the extreme environment.

## Author contributions

LS: Writing – review & editing, Writing – original draft. GZ: Writing – review & editing. TM: Writing – review & editing, Writing – original draft. WK: Writing – review & editing. BD: Writing – review & editing.
